# Astragaloside IV Attenuates Ocular Hypertension in a Mouse Model of TGFβ2 Induced Primary Open Angle Glaucoma

**DOI:** 10.3390/ijms222212508

**Published:** 2021-11-19

**Authors:** Ramesh B. Kasetti, Prabhavathi Maddineni, Bindu Kodati, Bhavani Nagarajan, Sam Yacoub

**Affiliations:** Department of Pharmacology and Neuroscience and the North Texas Eye Research Institute, University of North Texas Health Science Center, Fort Worth, TX 76107, USA; prabhavathi.maddineni@unthsc.edu (P.M.); Bindu.Kodati@unthsc.edu (B.K.); bhavani.nagarajan@unthsc.edu (B.N.); sam.yacoub@unthsc.edu (S.Y.)

**Keywords:** astragaloside IV, trabecular meshwork, TGFβ2, myocilin, ECM, MMP, ER stress, POAG, glaucoma

## Abstract

Elevated intraocular pressure (IOP) is a major risk factor in developing primary open angle glaucoma (POAG), which is the most common form of glaucoma. Transforming growth factor-beta 2 (TGFβ2) is a pro-fibrotic cytokine that plays an important role in POAG pathogenesis. TGFβ2 induced extracellular matrix (ECM) production, deposition and endoplasmic reticulum (ER) stress in the trabecular meshwork (TM) contribute to increased aqueous humor (AH) outflow resistance and IOP elevation. Drugs which alter the glaucomatous fibrotic changes and ER stress in the TM may be effective in reducing ocular hypertension. Astragaloside IV (AS.IV), a novel saponin isolated from the roots of *Astragalus membranaceus*, has demonstrated antifibrotic and ER stress lowering effects in various tissues during disease conditions. However, the effect of AS.IV on glaucomatous TM fibrosis, ER stress and ocular hypertension has not been studied. Primary human TM cells treated with AS.IV decreased TGFβ2 induced ECM (FN, Col-I) deposition and ER stress (KDEL, ATF4 and CHOP). Moreover, AS.IV treatment reduced TGFβ2 induced NF-κB activation and αSMA expression in TM cells. We found that AS.IV treatment significantly increased levels of matrix metalloproteases (MMP9 and MMP2) and MMP2 enzymatic activity, indicating that the antifibrotic effects of AS.IV are mediated via inhibition of NF-κB and activation of MMPs. AS.IV treatment also reduced ER stress in TM3 cells stably expressing mutant myocilin. Interestingly, the topical ocular AS.IV eye drops (1 mM) significantly decreased TGFβ2 induced ocular hypertension in mice, and this was associated with a decrease in FN, Col-1 (ECM), KDEL (ER stress) and αSMA in mouse TM tissues. Taken together, the results suggest that AS.IV prevents TGFβ2 induced ocular hypertension by modulating ECM deposition and ER stress in the TM.

## 1. Introduction

Primary open angle glaucoma (POAG) is the most common type of glaucoma, accounting for nearly 74% of all glaucoma cases [1,2]. Elevated intraocular pressure (IOP) is the only modifiable risk factor associated with POAG [3]. The balance between aqueous humor (AH) secretion from the ciliary body and its outflow through the trabecular meshwork (TM) are physiologically important to maintain normal IOP. In POAG, there is increased resistance to aqueous humor outflow through the TM, leading to an elevation of IOP [4,5]. Increased synthesis and deposition of extracellular matrix (ECM) proteins in the TM (TM fibrosis) are responsible for TM dysfunction and the subsequent increase in AH outflow resistance [6,7,8,9]. Apart from ECM deposition, our studies have shown that ER stress also plays a major role in glaucomatous TM dysfunction [10,11,12,13,14,15]. The endoplasmic reticulum (ER) is involved in the synthesis, folding, and trafficking of proteins in eukaryotic cells. Pathology associated with disease conditions such as protein misfolding (myocilin-associated glaucoma) or increased synthesis (steroid or TGFβ2-induced glaucoma) can disturb protein homeostasis in the ER, resulting in ER stress. ER stress and ECM deposition are interlinked through signaling mechanisms. Chronic ER stress disturbs ER homeostasis affecting the processing and secretion of ECM proteins, leading to abnormal ECM accumulation in the TM [9]. Conversely, increased synthesis of ECM proteins overloads the ER processing capacity, which leads to induction of ER stress and ultimately to abnormal ECM accumulation in the TM [15]. Current glaucoma therapeutics are effective in reducing IOP, but none address the underlying pathological mechanisms of TM dysfunction in POAG such as ECM accumulation and ER stress. Thus, targeting ECM accumulation and ER stress in the TM may provide a novel treatment that mitigate disease progression in the treatment of glaucoma.

Several factors including TGFβ2 are known to contribute to glaucomatous IOP elevation. TGFβ2 levels are elevated in aqueous humor samples and TM tissues of POAG patients [16,17,18,19]. TGF-β2 induces epithelial-to-mesenchymal transition (EMT-like) changes in TM cells, such as increased ECM production, actin stress fiber formation and alpha smooth muscle actin (α-SMA) expression [20,21,22]. Treatment of primary human TM cells with TGFβ2 increases synthesis and deposition of ECM [20,23]. Adenoviral injections of bioactive TGFβ2 have been shown to elevate IOP and induce ECM deposition in mouse TM tissues [24,25]. In addition, TGFβ2 treatment elevated IOP and increased ECM deposition in TM tissues of the ex-vivo human anterior segment perfusion culture model [20,26]. Recently we have demonstrated that TGFβ2 treatment induces ECM deposition and ER stress in primary human TM cells and TM tissues of ex-vivo cultured human corneo-scleral segments [11]. TGFβ2 induced ocular hypertension was found to be associated with induction of ER stress in mouse TM tissues (manuscript under preparation, Patil et al., 2021). These studies show that increased ECM deposition and ER stress are important players in TGFβ2 induced ocular hypertension.

Pharmacological agents that modulate ECM synthesis, composition and ER stress in the TM have been shown to reduce ocular hypertension. Li et al. demonstrated that netarsudil, a rho-kinase inhibitor, decreased glucocorticoid-induced ocular hypertension by decreasing TM tissue fibrosis [27]. The current IOP lowering drugs, prostaglandin analogs and beta-blockers, modulate ECM composition in the ciliary body via activation of MMPs [28,29,30]. We and others have shown that mTOR inhibitors effectively reduce glaucomatous IOP elevation and are associated with a reduction in TM fibrosis and ER stress [31,32]. Zode et al. have reported that topical ocular phenylbutyric acid (PBA) treatment rescues mutant myocilin associated and steroid induced ocular hypertension by decreasing ER stress in the TM [33,34]. In addition, recent findings have demonstrated that PBA reduces ECM synthesis and deposition in the TM by increasing MMP9 activity [35]. Recently we have shown that ISRIB treatment (previously shown to reduce ER stress) effectively reversed steroid and mutant myocilin induced ocular hypertension [10]. These studies demonstrate the importance of discovering new pharmacological agents that simultaneously target glaucomatous TM fibrosis and ER stress for the treatment of glaucoma.

**Figure 1:**
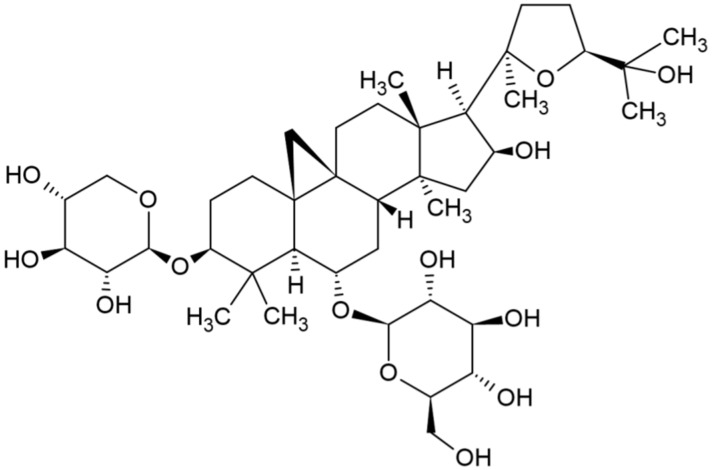
Chemical structure of astragaloside IV. A pentacyclic triterpenoid that is cycloastragenol having β-D-xylopyranosyl and β-D-glucopyranosyl residues attached at positions O-3 and O-6 respectively (3-O-β-D-xylopyranosyl-6-O-β-D-glucopyranosyl-cycloastragenol). C_14_H_68_O_14_.

Natural compounds derived from medicinal plants are becoming an important source for the development of new drugs as they exhibit minimal side effects [36]. Astragaloside-IV (AS.IV, Figure 1) is a novel saponin isolated from the roots of *Astragalus membranaceus*, a commonly used Chinese medicinal herb. It has been shown to have several therapeutic effects and a good safety profile compared with other natural products, making it a promising lead compound for drug discovery. AS.IV exhibited antifibrotic activity in animal models of disease including renal, hepatic, and myocardial fibrosis [37,38,39]. A number of studies have examined the ER stress lowering activity of AS.IV in several disease models [40,41,42,43] and found it is mainly mediated by inhibiting the ATF4-CHOP pathway [44]. Ongoing clinical studies have revealed that administration of AS.IV is safe, making it an attractive drug [45,46]. To date, there is no study that has evaluated the role of AS.IV in glaucoma. Based on the recent findings demonstrating the antifibrotic and ER stress lowering effects of AS.IV, we hypothesized that AS.IV may reduce fibrotic changes and reduce ER stress in the TM and consequently lower IOP. In the present study, we found that AS.IV treatment lowered TGFβ2 induced ocular hypertension in mice and is further associated with decreased ECM deposition and ER stress in the TM.

## 2. Results

### 2.1. Astragaloside IV Treatment Attenuates the TGFβ2 Induced Fibrotic Changes and ER Stress in TM Cells

**Figure 2:**
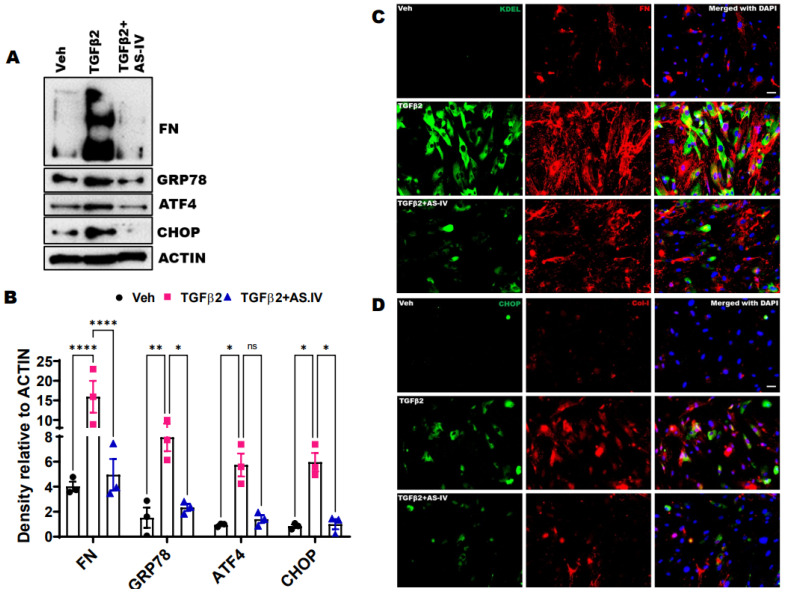
AS.IV treatment prevents the TGFβ2 induced ECM deposition and ER stress in TM cells. The primary human TM cells were treated with vehicle, TGFβ2 (5 ng/mL) and TGFβ2 plus AS.IV (100 µM) for 3 days. (**A**,**B**) The cell lysates were analyzed by Western blot (**A**) and densitometric (**B**) analysis of ECM (FN) and ER stress (GRP78, ATF4, and CHOP) markers. The AS.IV co-treatment significantly reversed the TGFβ2 induced ECM changes and ER stress. (*n* = 3 cell strains, data represented as mean ± SEM, 2-way ANOVA, Tukey’s multiple comparisons test, * *p* < 0.05, ** *p* < 0.01, **** *p* < 0.0001). (**C**,**D**) represents immunostaining analysis on fixed TM cells treated with vehicle, TGFβ2 and TGFβ2 plus AS.IV. A prominent decrease in FN, Col-I (ECM), KDEL and CHOP (ER stress) staining in the AS.IV co-treated cells compared to the TGFβ2 treatment alone, indicates decreased ECM deposition and ER stress (*n* = 3 cell strains, scale bar 10 µm).

Previous studies have demonstrated the anti-fibrotic and ER stress lowering activities of AS.IV in different cell types and tissues [37,38,39,40,41,42,43,44]. Here, we have examined whether AS.IV treatment prevents TGFβ2 induced ECM deposition and ER stress in primary human TM cells (*n* = 3 cell strains). Western blot analysis revealed that TGFβ2 treatment significantly increases ECM deposition and ER stress induction as shown by increased ECM (FN) and ER stress (GRP 78, ATF4 and CHOP) markers compared to the vehicle (Veh) treated control. TGFβ2 induced fibrotic response and ER stress are significantly suppressed by AS.IV co-treatment (at 100 µM concentration) (Figure 2A,B). Similarly, immunostaining revealed that TGFβ2 treatment increased the FN, Col-I (ECM markers), KDEL and CHOP (ER stress markers) staining in primary human TM cells compared to vehicle treated control, and AS.IV co-treatment effectively reduced the staining intensities of both ECM and ER stress markers (Figure 2C,D). These studies have clearly demonstrated that AS.IV significantly reduces the TGFβ2 induced ECM deposition and ER stress in TM cells. However, AS.IV treatment did not show any effect on TGFβ2 induced ECM changes and ER stress at the lower 50 µM dose (Figure S1).

### 2.2. Astragaloside IV Treatment Inhibits TGFβ2 Induced NF-κB Activation and αSMA Expression in TM Cells

**Figure 3:**
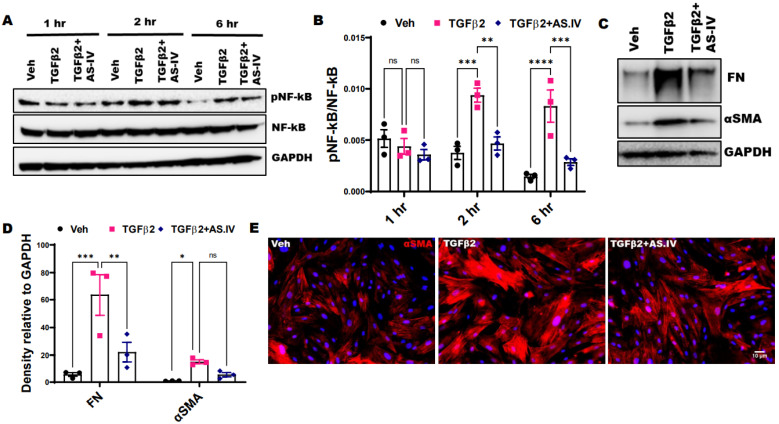
AS.IV treatment attenuates TGFβ2 induced NF-κB activation and αSMA expression. (**A**,**B**) Western blot (**A**) and its densitometric (**B**) analysis of TM3 cell lysates treated with vehicle, TGFβ2 (5 ng/mL) and TGFβ2 plus AS.IV (100 µM) at various time points (1, 2 and 6 h). The TGFβ2 significantly increased pNF-κB/NF-κB levels at 2 & 6 h after the treatment, indicating the activation of NF-κB signaling. The AS.IV co-treatment significantly suppressed the TGFβ2 induced NF-κB activation (*n* = 3 replicates, data represented as mean ± SEM, 2-way ANOVA, Tukey’s multiple comparisons test, ** *p* < 0.01, *** *p* < 0.001, **** *p* < 0.0001). (**C**–**E**) Western blot (**C**), its densitometric (**D**) and immunostaining (**E**) analysis of primary human TM cells treated with vehicle, TGFβ2 (5 ng/mL) and TGFβ2 plus AS.IV (100 µM) for 3 days. The TGFβ2 treatment significantly increased the αSMA expression whereas AS.IV co-treatment prominently suppressed. Scale bar 10 µm. (*n* = 3 cell strains, data represented as mean ± SEM, 2-way ANOVA, sidak’s multiple comparisons test, * *p* < 0.05, ** *p* < 0.01, *** *p* < 0.001).

The activation of NF-κB signaling plays an important role in TGFβ2 induced ECM production and ocular hypertension [47]. Previous studies have demonstrated the inhibitory effect of AS.IV against NF-κB activation in various cell types and tissues [48,49,50]. Here, we have examined the effect of TGFβ2 and AS.IV on NF-κB activation in TM cells. The primary human TM cells (*n* = 3 different cell strains) were treated with Vehicle, TGFβ2 (5 ng/mL) and TGFβ2 (5 ng/mL) plus AS.IV (100 µM) at different time points (1, 2 and 6 h) and the cell lysates were analyzed for pNF-κB and NF-κB levels by Western blot and densitometry (Figure 3A,B). TGFβ2 treatment activated NF-κB signaling (increased pNF-κB/NF-κB or pNF-κB/GAPDH levels compared to vehicle control) as early as 2 h after the treatment. AS.IV co-treatment suppressed the TGFβ2 induced NF-κB activation, as evident by significantly decreased pNF-κB/NF-κB levels compared to TGFβ2 treatment alone. The inhibitory effect of AS.IV on TGFβ2 induced NF-κB activation was more prominent at 6 h compared to 2 h after the treatment (Figure 3A,B and Figure S2). In consistence with previous studies, the TGFβ2 treatment significantly increased αSMA levels in primary human TM cells as shown in Western blot and densitometric analysis [10,20,51] (Figure 3C,D). AS.IV co-treatment reduced αSMA levels, however this reduction is not statistically significant (*p* = 0.07051) (Figure 3D). This may be due to variability in AS.IV action against three different cell strains collected from three different donors. In contrast to the Western blot, immunostaining analysis revealed that AS.IV co-treatment effectively reduced the αSMA staining against TGFβ2 induction (Figure 3E).

### 2.3. Antifibrotic Effects of AS.IV Are Mediated via Increased Metalloproteases Activity in TM Cells

**Figure 4:**
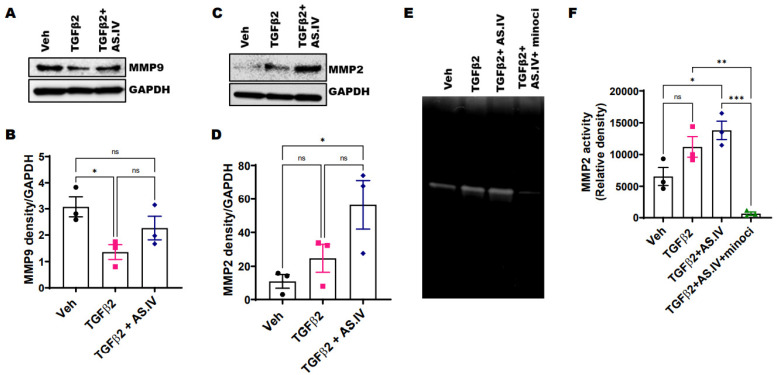
AS.IV treatment enhances the MMPs activity in the TM cells. (**A**–**D**) The primary human TM cells were treated with vehicle, TGFβ2 (5 ng/mL) and TGFβ2 plus AS.IV (100 µM) for 3 days and the cell lysates were subjected to Western blot (**A**,**C**) and its densitometric (**B**,**D**) analysis of MMP9 and MMP2 (*n* = 3 cell strains, data represented as mean ± SEM, One-way ANOVA, Tukey’s multiple comparisons test, * *p* < 0.05). TGFβ2 treatment resulted in a significant reduction of MMP9 levels and a slight increase in MMP2 levels. AS.IV co-treatment prominently increased both MMP9 and MMP2 levels. (**E**,**F**) The primary human TM cells were treated with vehicle, TGFβ2 (5 ng/mL), TGFβ2 plus AS.IV (100 µM) and TGFβ2 + AS.IV (100 µM) + minocycline (200 µM) for 3 days in a serum free medium and spent medium was concentrated and subjected to the gelatin zymography (**E**). The densitometric analysis (**F**) of the gelatinolytic bands corresponding to MMP2 activity revealed that AS.IV treatment significantly increased the MMP2 activity and this was completely inhibited by minocycline (minoci) treatment, a PAN MMPs inhibitor (*n* = 3 cell strains, data represented as mean ± SEM, One-way ANOVA, Tukey’s multiple comparisons test, * *p* < 0.05, ** *p* < 0.01, *** *p* < 0.001, ns—not significant).

Matrix metalloproteases (MMPs) are important modulators of aqueous humor outflow and IOP by continuously remodeling the TM extracellular matrix composition. Previous studies have demonstrated that ocular hypertension leads to changes in the expression and activity of several MMPs in the TM [52]. In the present study, Western blot and its densitometric analysis of the primary human TM cell lysates treated with TGFβ2 showed a significant decline in MMP9 expression compared to vehicle treated control (Figure 4A,B). Conversely, TGFβ2 treatment slightly increased MMP2 expression levels compared to vehicle, but this increase is not statistically significant (Figure 4C,D). AS-IV co-treatment restored the MMP9 levels to near vehicle control (Figure 4A,B) and significantly increased MMP2 levels compared to vehicle (Figure 4C,D). We further evaluated the effects of TGFβ2 and AS.IV on the enzyme activities of MMPs by conducting a gelatin zymography (Figure 4E,F). The primary human TM cells were treated with vehicle, TGFβ2 (5 ng/mL), TGFβ2 + AS.IV (100 µM) and TGFβ2 + AS.IV + minocycline (100 µM) for 3 days in serum free condition and the spent medium was concentrated and subjected to gelatin zymography (Figure 4E). Surprisingly, the densitometric analysis of zymography (Figure 4F) revealed that both TGFβ2 and AS.IV co-treatments increased MMP2 enzymatic activity as evident from the increased MMP2 gelatinolytic band intensity compared to the vehicle control. However, this increase in enzymatic activity was statistically significant only with AS.IV co-treatment (*p* = 0.0181) but not with TGFβ2 treatment alone (*p* = 0.1300). The minocycline (a pan-MMPs inhibitor) co-treatment significantly inhibited the MMP2 enzymatic activity compared to AS.IV co-treatment, act as a positive control. Unfortunately, we did not detect the corresponding MMP9 gelatinolytic bands in gelatin zymography. These data indicates that AS.IV regulate ECM composition by modulating MMPs levels and their activities in the TM cells.

### 2.4. Astragaloside IV Treatment Effectively Decreases Mutant Myocilin Associated ER Stress in the TM Cells

**Figure 5:**
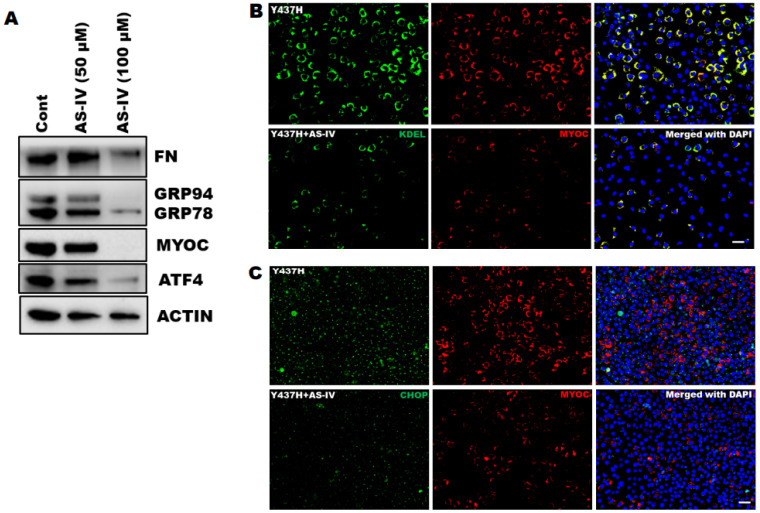
AS.IV treatment prevents mutant myocilin associated ER stress in TM cells. (**A**) TM3 cells stably expressing DsRed tagged mutant myocilin (DsRed-MYOC^Y437H^) were treated with AS.IV (50 and 100 µM) for 3 days. The cell lysates were analyzed by Western blot using ER stress markers (KDEL that recognizes GRP78 & GRP94, ATF4), ECM marker (FN) and myocilin. The AS.IV at 100 µM concentration effectively reduced mutant myocilin induced ER stress and that is further associated with reduction in FN and intracellular accumulation of mutant myocilin (*n* = 2 replicates). (**B**,**C**) TM3 cells stably expressing DsRed-MYOC^Y437H^ were treated with AS.IV (100 µM) for 3 days. The cells were fixed and stained for KDEL (**B**) and CHOP (**C**). AS.IV treatment prominently reduced KDEL and CHOP staining which is associated with decreased DsRed expression in the cells indicating the reduction in the mutant myocilin accumulation (*n* = 3 replicates). Scale bar 50 µm.

Myocilin (MYOC) mutations resulting in elevated IOP are responsible for approximately 4% of POAG and most cases of autosomal dominant juvenile-onset-open-angle glaucoma [4,53]. We have previously reported that expression of mutant myocilin (Y437H) leads to accumulation inside the ER of the TM cells and triggers ER stress [9,33]. In this study, we utilized this in vitro model (mutant myocilin induced ER stress) to verify the ER stress lowering activity of AS.IV. TM3 cells stably expressing DsRed tagged mutant myocilin (Y437H) were treated with vehicle and AS.IV (50 and 100 µM concentration) and lysates were subjected to Western blot analysis of ER stress markers (Figure 5A). AS.IV treatment at 100 µM concentration effectively reduced mutant myocilin associated ER stress as evident from a prominent decrease in GRP78, GRP94 and ATF4 levels compared to the control. Interestingly, AS.IV mediated ER stress reduction is associated with decreased FN and mutant myocilin accumulation in the TM cells. AS.IV treatment at 50 µM concentration also decreased mutant myocilin accumulation and ER stress but this decrease is not as prominent as 100 µM concentration. Similarly, immunostaining analysis also revealed that AS.IV (100 µM) treatment effectively reduced the DsRed (represents mutant myocilin), KDEL and CHOP expression levels compared to the control (Figure 5B,C), indicating that AS.IV mediated reduction of ER stress prevents mutant myocilin accumulation in the TM cells.

### 2.5. Astragaloside IV Treatment Reversed the TGFβ2 Induced Ocular Hypertension in the Mice

**Figure 6:**
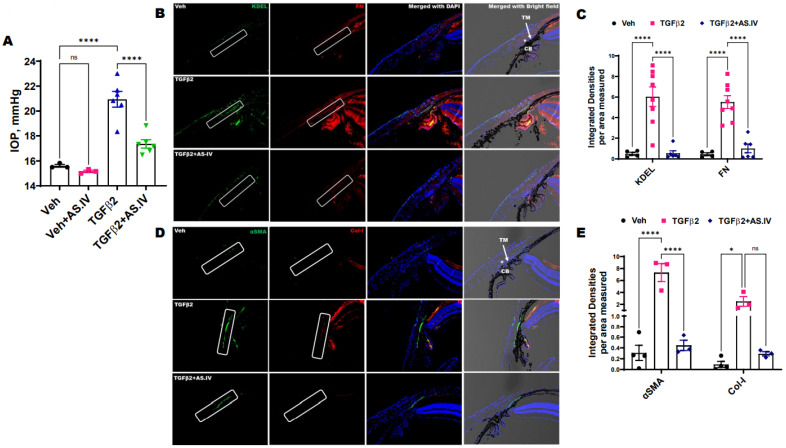
Topical ocular AS.IV treatment attenuates the TGFβ2 induced ocular hypertension in mice. The C57BL/6J mice (3–4 months age) eyes were injected with adenoviral vectors expressing bioactive form of TGFβ2 or Null intravitreally. After 3 weeks following injections, both groups received 5 µL of 1 mM AS.IV topical eye drops in the left eyes and vehicle eye drops (DMSO) in the contralateral right eyes twice daily. (**A**) One week of AS.IV treatment significantly lowered IOPs in TGFβ2-injected left eyes compared to the contralateral right eyes (*n* = 3–6 biologically independent samples, data represented as mean ± SEM, Two-way ANOVA, Tukey’s multiple comparisons test, **** *p* < 0.0001). (**B**–**E**) Immunostaining (**B**,**D**) on paraffin sectioned mouse eyes from the groups and their densities (**C**,**E**) revealed that AS.IV treatment significantly decreased the staining intensities of αSMA, ECM and ER stress markers in TM tissues of Ad5.TGFβ2 injected eyes. Scale bar 50 µM, TM-trabecular meshwork indicated with an arrow and rectangle box, CB- ciliary body, Schlemm’s canal indicated with an asterisk (*n* = 3–8 biological replicates, data represented as mean ±SEM, Two-way ANOVA, Tukey’s multiple comparisons test, * *p* < 0.05, **** *p* < 0.0001, ns—not significant).

Next, we examined whether topical ocular AS.IV eye drops lower IOP in a mouse model of TGFβ2 induced ocular hypertension. Three-month old C57 mice were divided into two groups, and one group received a single intravitreal injection of Ad5.CMV.TGFβ2 (1 × 10^7^ pfu/eye) while the other group was injected with Ad5.CMV.Null (1 × 10^7^ pfu/eye) vectors bilaterally. Two weeks post injection, a significant IOP elevation was observed in the Ad5.TGFβ2 injected group compared to the Ad5.null injected group. After confirming the TGFβ2 induced OHT, both groups received AS.IV (1 mM) topical ocular eye drops twice a day for 2 weeks in one eye and vehicle eye drops (0.1% DMSO) in the contralateral eye. The IOPs were recorded every week for 2 weeks. Within a week, AS.IV treated eyes showed significantly decreased IOPs (16.58 ± 0.594) compared to the vehicle treated contralateral eyes (20.5 ± 0.436) in the Ad5.TGFβ2 group (Figure 6A). Similarly, the Ad5.Null group treated with AS.IV also showed a slight reduction in IOPs (14.27 ± 0.146) compared to the vehicle treated contralateral eyes (15.77 ± 0.111), however the reduction was not statistically significant (*p* = 0.8224). The AS.IV effect on TGFβ2 induced IOP elevation was similar in both the 1st week and in 2nd week after treatment (Figure S3).

The immunostaining and density analysis of ECM and ER stress markers (FN, Col-I, KDEL), and the expression of αSMA in the eye sections from the above groups revealed that Ad5.TGFβ2 lead to a significant increase in FN, KDEL (Figure 6B,C) and Col-I, αSMA (Figure 6D,E) expression levels in the TM region compared to the Ad5.Null (vehicle) group. However, treatment with AS.IV reversed the TGFβ2 induced changes as evident from the significant reduction of FN, KDEL and αSMA staining compared to the TGFβ2 group. Similarly, AS.IV treatment decreased the Col-I staining, but was not statistically significant (*p* = 0.0760). This may be due to a low sample size (*n* = 3) that can be resolved by increasing the number of samples to achieve a statistically significant reduction. These data indicate that AS.IV treatment modulates TGFβ2 induced ECM deposition and ER stress, thereby decreasing ocular hypertension.

## 3. Discussion

TM dysfunction and the associated increase in aqueous humor (AH) outflow resistance contribute to IOP elevation in POAG. Lowering IOP remains the only therapeutic approach for preserving visual function in glaucoma patients. The majority of IOP lowering drugs do not target TM pathology to improve trabecular meshwork AH outflow [54]. Therefore, there is an unmet need to identify novel drugs that can prevent or reverse TM dysfunction by targeting the underlying molecular mechanisms. Evidence has suggested that increased synthesis and deposition of ECM (TM fibrosis) and induction of ER stress in the TM are associated with TM dysfunction in glaucoma [6,7,8,9,10,11,12,13,14,15]. The drugs that target TM fibrosis or ER stress are effective in lowering IOP [10,27,31,32,33,34,35]. Based on previous literature, we found that AS.IV, a natural saponin exhibited both antifibrotic and ER stress lowering activities in various diseases [37,38,39,40,41,42,43,44]. We hypothesized that AS.IV can effectively mitigate the TGFβ2 induced OHT by modulating ECM deposition and ER stress in the TM. In support of our hypothesis, and demonstrated in this study, topical ocular AS.IV treatment significantly decreased TGF-β2 induced ocular hypertension in mice. It is possible that the IOP lowering activity of AS.IV was mediated via reduction of ER stress and ECM deposition in the TM, as evident from significantly decreased ECM and ER stress markers in the AS-IV treated TM tissues. Although we have not evaluated AS.IV drug penetration through the ocular tissues, the pharmacological changes observed in the AS.IV treated group indicates that AS.IV can reach the TM to exert its pharmacological action.

The rationale behind selection of the TGFβ2 induced OHT model over the other conventional models is multifactorial. The pathological role of TGFβ2 in POAG has been extensively studied. Additionally, increased levels of TGFβ2 have been reported in the aqueous humor and TM tissues of POAG patients [16,17,18,19]. Expression of bioactive TGFβ2 has been found to elevate IOP in mice [24,25]. Moreover, TGFβ2 induced OHT resembles the disease phenotype seen in POAG patients. Similar to POAG, TGFβ2 treatment increases ECM synthesis and deposition in the TM that is associated with induction of ER stress [11,20,26]. TGFβ2 levels (pro and active) are elevated both in steroid-induced glaucoma [14] and myocilin-associated glaucoma [55], suggesting that TGFβ2 signaling is an important mediator in OHT models.

Inconsistent with the previous report [47], TGFβ2 treatment significantly enhanced NF-kB activation in the TM cells and is necessary for TGFβ2 induced ECM production and ocular hypertension. It has been shown that AS.IV suppresses TGFβ1 induced renal fibrosis by inhibiting NF-kB activation [56,57]. Similarly, AS.IV treatment significantly suppressed the TGFβ2 mediated activation of NF-kB in TM cells, suggesting that the antifibrotic effects of AS.IV are mediated via inhibition of NF-kB signaling. The exact mechanism involving the inhibition of NF-kB in preventing TGFβ2 induced ECM deposition in the TM cells is unknown. However, it is known that the activation of NF-kB downregulates BAMBI, a known endogenous inhibitor of TGFβ signaling (via inhibition of SMAD3 activation) [58,59,60,61]. It is possible that AS.IV mediated inhibition of NF-kB signaling promotes BAMBI expression that directly inhibits canonical TGFβ2 signaling (SMAD3 dependent) and TM fibrosis.

In addition, the other important regulators of ECM turnover in tissues are matrix metalloproteinases (MMPs). Reduced activity of MMPs is involved in increased ECM deposition in glaucomatous TM tissues [62,63,64], whereas increasing MMPs’ activity in perfused human anterior segment organ cultures increased outflow facility [64]. Although several MMPs are expressed in the TM, MMP-2 (gelatinase A) and MMP-9 (gelatinase B) are the most prominent and have been well studied. Hence, we have examined the effects of TGFβ2 and AS.IV on the levels and activities of MMP2 and MMP9 in the TM cells. Consistent with the previous report [65], primary human TM cells treated with TGFβ2 slightly increased the expression and enzymatic activity of MMP2. In contrast, the expression levels of MMP9 were significantly decreased. Increased MMP2 activity might be due to a feedback response of the TM cells to TGFβ2 induced ECM deposition. Bradley et al. (2001) have reported that activity of MMP2 increases against TM mechanical stretch within 24–72 h, while activities of other MMPs remain unchanged [66], indicating that MMP2 is the initial responder against TM fibrosis induced by TGFβ2. However, this slightly increased MMP2 activity may not be sufficient to minimize TM fibrosis, under the conditions of sustained ECM deposition and significantly reduced MMP9 levels. AS.IV co-treatment with TGFβ2 significantly increased the expression and activity of MMP2 and restored the MMP9 levels to the level of the vehicle-treated group. A recent study has documented that MMP9 knockout mice exhibit an aberrant increase in ECM deposition, reduced AH outflow, and early onset ocular hypertension, suggesting that AS.IV mediated increase in MMP9 levels are critical to effectively controlling ECM turnover in the TM [67]. It should be noted that we did not observe the corresponding MMP9 gelatinolytic bands in zymography gels, and this may be due to the low basal expression of MMP9 in the TM cells compared to MMP2 [62].

Apart from ECM deposition, reorganization of the actin cytoskeleton in the TM is known to contribute to TM stiffness and IOP elevation [68,69]. The role of TGFβ2 in actin cytoskeletal remodeling in the TM is well studied. TGFβ2 has been shown to increase α-SMA expression and its incorporation into the actin stress fibers [10,20,51]. Consistent with this, primary human TM cells treated with TGFβ2 showed a significant increase in α-SMA expression. AS.IV co-treatment reversed TGFβ2 induced α-SMA expression in TM cells. It is possible that AS.IV directly inhibits TGFβ2 mediated SMAD3 activation and thereby controls the α-SMA expression in TM cells, as shown in other cell types and tissues [39,70].

ER stress is another important player in glaucoma pathogenesis. Chronic or sustained ER stress can initiate cell death via the pro-apoptotic ATF4-CHOP pathway. The chronic ER stress-inducible pro-apoptotic markers ATF4 and CHOP are significantly increased in mouse models of glaucoma and in TM tissues from POAG donors [9,11,12,71]. Adenoviral expression of ATF4 in the TM elevates IOP in WT mice and is associated with increased fibrotic changes in the TM [10]. Genetic or pharmacological inhibition of ATF4-CHOP pathway rescued ocular hypertension in mice, which is associated with decreased ECM deposition and ER stress in the TM [10]. In this context, AS.IV exhibited ER stress lowering activity in several disease models by targeting ATF4-CHOP pathway [44,72,73]. Similarly, in this study AS.IV reduced TGF-β2 and mutant myocilin induced ATF4 and CHOP levels, which is further associated with a reduction in ER stress and ECM accumulation in the TM.

In conclusion, our data provides new evidence that AS.IV rescues TGF-β2 induced ocular hypertension by modulating ECM deposition and ER stress in the TM. Our results support AS.IV as a natural compound that may be useful for the prevention or treatment of glaucoma. We believe that the molecular basis of AS.IV therapeutic efficacy may not be limited to the inhibition of one or two pathways as described in this study. The efficacy may result from a combination of several pathways, which warrants further investigation. The ongoing clinical studies have revealed that administration of AS.IV is safe in humans. However, in this study AS.IV exhibited pharmacological actions at a higher dose both in vitro (100 µM) and in vivo (1 mM). It is reported that saponins over an extended period at a high dose may exhibit some toxic side effects. We did not observe side effects either in vitro (such as cell death) nor in mice receiving 1mM topical ocular AS.IV. It should be noted that these studies are of short duration. An investigation of AS.IV safety on long-term use is recommended.

## 4. Materials and Methods

### 4.1. Antibodies and Reagents

Antibodies and reagents were purchased from the following sources: fibronectin (catalog # Ab2413, Abcam, Boston, MA, USA), collagen I (catalog # NB600-408, Novus Biologicals, Centennial, CO, USA), KDEL (catalog # Ab12223, Abcam), ATF4 (catalog # SC-200, Santa Cruz Biotechnology, Dallas, TX, USA), CHOP (catalog # 13172, Novus Biologicals), αSMA (catalog # ab5694, Abcam), pNF-κB p65 (S536) (catalog #3031S, Cell Signaling Technology, Danvers, MA, USA), NF-κB p65 (D14E12) (catalog #8242T, Cell Signaling Technology), MMP9 (N2C1) (catalog #100458, Genetex, Irvine, CA, USA), MMP2(catalog #104577, Genetex), myocilin (catalog # ab41552, Abcam), GAPDH (catalog # 3683, Cell Signaling Technology), and β-actin (catalog # 4970, Cell Signaling Technology). Recombinant human TGFβ2 (catalog # 302-B2-010, R&D systems), astragaloside IV (catalog # 12069, Cayman Chemicals, Ann Arbor, MI, USA). Adenoviral vectors expressing bioactive TGFβ2 or null were purchased from the University of Iowa viral vector core facility (Iowa City, IA, USA).

### 4.2. TM Cell Culture and Treatments

Primary human TM cell strains (*n* = 3) & transformed TM3 cells were cultured in DMEM-low glucose medium (Sigma, St. Louis, MO, USA) supplemented with 10% fetal bovine serum (Atlas Biologicals, Fort Collins, CO, USA), L-glutamine (Gibco, Life Technologies, Grand Island, NY, USA), and penicillin-streptomycin (Gibco, Life Technologies). For the characterization of primary human TM cells, cells were examined for the expression of fibronectin, collagen, and laminin as well as dexamethasone induction of cross-linked actin networks and myocilin as described previously [74]. Different set of treatments were performed as mentioned below: (i) Human primary TM cells (*n* = 3) were treated with either vehicle (4 mM HCl containing 0.1% BSA) or recombinant human TGFβ2 (5 ng/mL) or TGFβ2 plus AS.IV (50 and 100 µM) in 0.5% FBS containing DMEM medium for 3 days. (ii) To examine the effect of AS.IV on TGFβ2 induced NF-κB activation, transformed TM3 cells were treated with either vehicle (4 mM HCl containing 0.1% BSA) or recombinant human TGFβ2 (5 ng/mL) alone or in combination with AS.IV (100 µM) at different time points (1, 2 and 6 h). (iii) To examine the effect of AS.IV on mutant myocilin (Y437H) induced ER stress, transformed TM3 cells stably expressing DsRed-tagged-MYOC^Y437H^ were generated by transient transfection with pDsRed2-MYOC^Y437H^ plasmid and selection of colonies using G418 antibiotics (0.6 mg/mL; Gibco, Life Technologies) [9,32]. These stable clones expressing mutant myocilin were maintained in DMEM-low glucose medium supplemented with 10% fetal bovine serum (Atlas Biologicals) and G418 antibiotics. The TM3 cells expressing DsRed-tagged-MYOC^Y437H^, cultured in the media mentioned above, were treated with or without AS.IV (50 and 100 µM) for 3 days. After the treatment, the cell lysates were collected for Western blot analysis, and fixed cells were utilized for immunostaining analysis.

### 4.3. Gelatin Zymography

To access the effect of AS.IV on the activity of gelatinases (MMP2 and MMP9), the primary human TM cells were treated with either vehicle (4 mM Hcl containing 0.1% BSA) or TGFβ2 (g/mL) or TGFβ2 (5 ng/mL) plus AS.IV (100 µM) for 3 days under serum-free conditions. The conditioned media was concentrated using pierce protein concentrators with 10 K MWCO (Catalog # 88513, ThermoFisher Scientific, Waltham, MA, USA). The concentrated conditioned media was subjected to gelatin zymography electrophoresis using Novex 10% Zymogram Plus (gelatin) Protein Gels (catalog #ZY00100BOX, ThermoFisher Scientific) under native conditions. Following electrophoresis, gels were incubated in zymogram renaturing buffer (catalog # LC2670, ThermoFisher Scientific) for 30 min at room temperature with gentle agitation. Then, gels were incubated with zymogram developing buffer (catalog #LC2671, ThermoFisher Scientific) for 12 h at room temperature. Then gels were stained with SimplyBlue Safestain (catalog # LC6060, ThermoFisher Scientific) at room temperature until clear bands were visible against a dark background.

### 4.4. Experimental Animals

All experimental procedures were conducted in accordance with and adherence to the ARVO Statement for the Use of Animals in Ophthalmic and Vision Research. The experimental protocol was approved by the Institutional Animal Care and Use Committee (IACUC) (Protocol #: IACUC-2018-0032) of the University of North Texas Health Science Center (UNTHSC). Three-month-old C57BL/6J mice purchased from the Jackson Laboratory (Bar Harbor, ME, USA) were utilized in this study. Animals were allowed to roam freely in their cages, have access to food (standard mouse chow) and water ad libitum, and were maintained under 12 h light/12 h dark conditions.

### 4.5. Adenoviral Injections

To generate TGFβ2 induced ocular hypertension in mice, adenoviral vector expressing bioactive form of TGFβ2 was injected intravitreally (1 × 10^7^ pfu/eye in 2 µL injection volume) in C57 mice as described earlier [24]. Intravitreal injection of Adenoviral null vector-alone served as control. The TGFβ2 induced ocular hypertension was confirmed by measuring IOP once a week. After confirming ocular hypertension, the mice were given topical ocular AS.IV eye drops twice a day for 2 weeks. The mice were euthanized and the eyes were enucleated, fixed in 4% PFA, and processed for paraffin sectioning and staining.

### 4.6. IOP Measurements

Daytime IOPs were measured using a TonoLab rebound tonometer (Colonial Medical Supply, Londonderry, NH, USA) between 9 am and 11 am under isoflurane anesthesia (isoflurane 2.5% and oxygen 0.8 L/min) as previously described [9,10,32,75]. Six individual IOP measurements were obtained in a masked manner and averaged to obtain the final IOP value for each eye. To avoid the influence of isoflurane, the IOP measurements were completed within 2–3 min.

### 4.7. Western Blot Analysis

Primary human TM cells or transformed human TM3 cells were lysed in RIPA lysis buffer as described previously [10,11,32]. Equal protein concentrations of lysates were loaded and run in denaturing 4–12% bis-Tris gels (NuPAGE bis-Tris gels, Life Technologies). The separated proteins in the gel were then transferred onto a polyvinylidene difluoride (PVDF) membrane. The blots were blocked in 10% nonfat dried milk prepared in 1xPBST (PBS with 0.1% Tween 20) for 2 h at room temperature and then incubated with the appropriate primary antibodies (1:1000) on a rotating shaker for overnight at 4 °C. The blots were washed three times with 1xPBST followed by a secondary antibody (horseradish peroxidase-conjugated) incubation for 1.5 h at room temperature. The blots were developed using ECL detection reagents (SuperSignal West Femto maximum sensitivity substrate, Life Technologies). For the detection of phosphorylated NF-κB, the blots were incubated with 3% BSA instead of 10% nonfat dried milk for blocking and antibody incubation.

### 4.8. Immunostaining

Enucleated mouse eyes were fixed in 4% paraformaldehyde (PFA) overnight, washed three times with 1xPBS, and dehydrated/immersed in 70% ethanol, processed and embedded in paraffin. Five-micron thin paraffin sections were prepared. For immunostaining of tissue sections, the slides were deparaffinized using two washes of xylene rehydrated with descending gradients of ethanol (100%, 90%, 70%, and 50%) and finally with 1× PBS. The slides were then subjected to antigen retrieval in 0.1 M citrate buffer (pH 6) for an hour in a water bath set at 60 °C. Slides were then blocked in 10% goat serum containing 0.1% Triton X-100 for 2 h at room temperature and incubated with respective primary antibodies (1:300 dilution) in blocking buffer overnight at 4 °C. Sections were washed 3 times with 1× PBS and incubated with appropriate Alexa Fluor secondary antibody (1:500 dilution; Invitrogen, Waltham, MA, USA) for two hours at room temperature. Slides were then washed 3 times with 1× PBS and mounted with DAPI mounting media (VECTASHIELD Antifade Mounting Medium, Vector Laboratories, Burlingame, CA, USA). Following the same procedure, sections without the primary antibody incubation were used as negative control. Images were taken using a fluorescence microscope (Keyence, Itasca, IL, USA). For immunostaining of TM cells, cells were fixed in 4% PFA for 15 min, permeabilized with 0.1% Triton X-100 in PBS for 10 min and stained with the appropriate antibodies as described above.

### 4.9. Statistical Analysis

All the data are presented as mean ±SEM. Statistical significance between two groups was analyzed using the unpaired 2-tailed Student’s *t*-test. For data between multiple groups, one-way or two-way ANOVA with Tukey’s multiple comparisons test was used. *p* ≤ 0.05 was considered statistically significant.

## Data Availability

All data that support the reported results are included in this manuscript and in the Supplementary Materials.

## Supplementary Materials

The following are available online at https://www.mdpi.com/article/10.3390/ijms222212508/s1.
