# Supplementary figures and images for: Astragaloside IV Attenuates Ocular Hypertension in a Mouse Model of TGFβ2 Induced Primary Open Angle Glaucoma
Source: Int J Mol Sci. 2021 Nov 19;22(22):12508. doi: 10.3390/ijms222212508 (PMC8619727; doi:10.3390/ijms222212508)

Figure S1

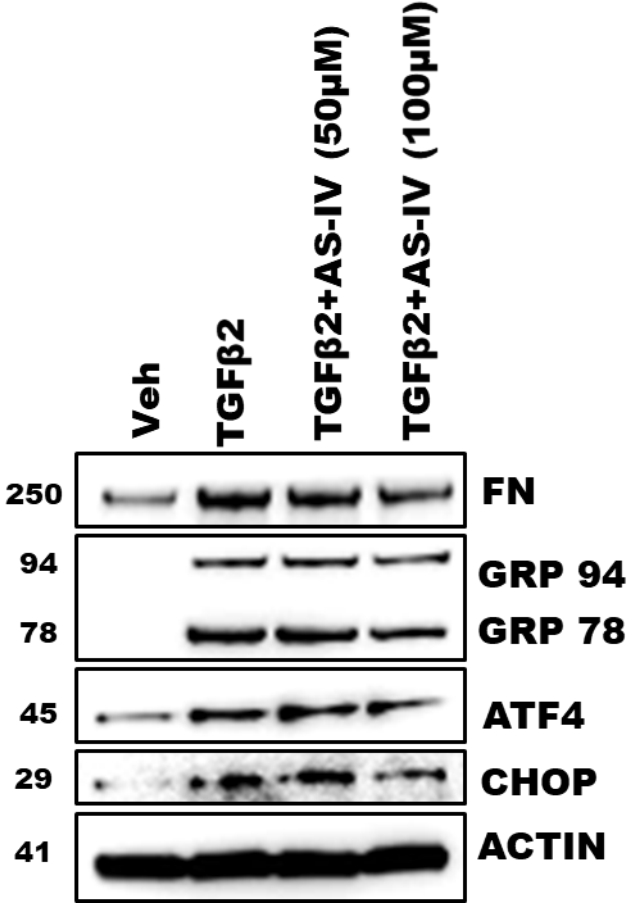

Figure S2

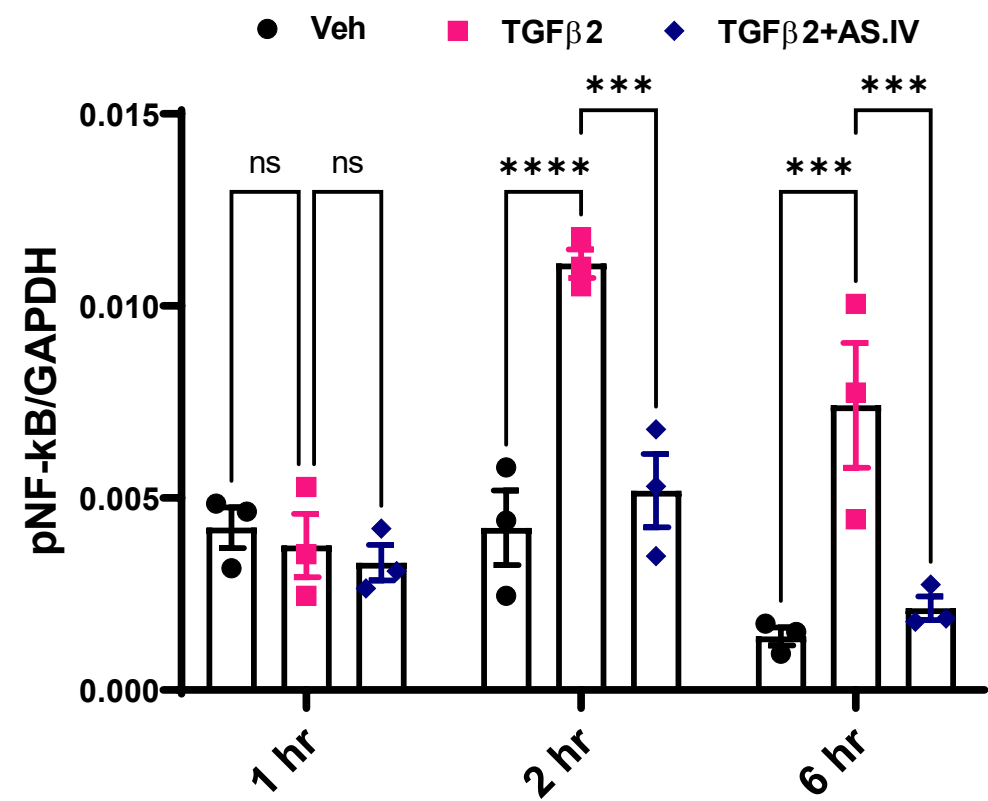

Figure S3

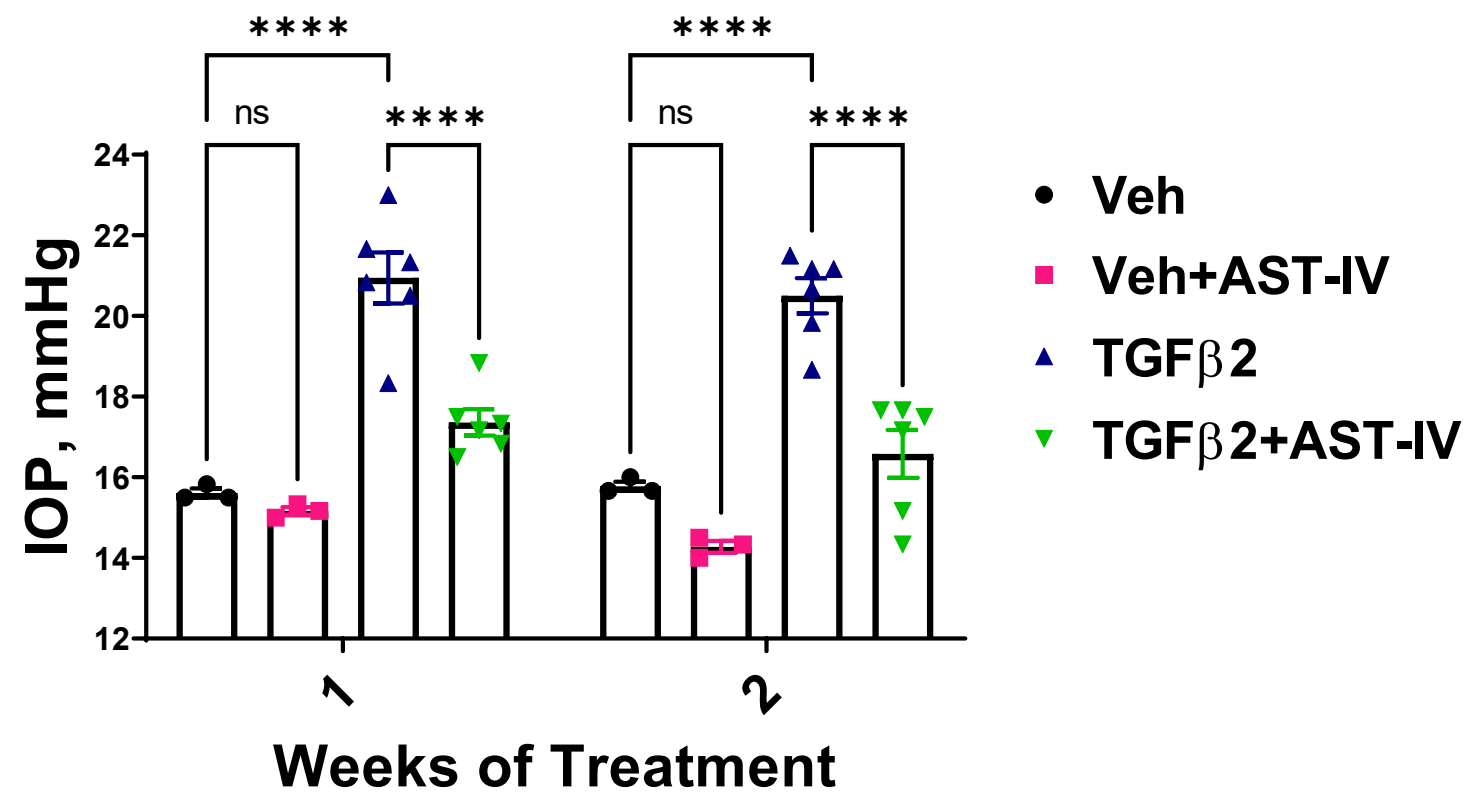

Supplement: Supplementary file 1 [file ijms-22-12508-s001.zip › ijms-1436068 -supplementary.pdf]
